# Fluorescent Organic Small Molecule Probes for Bioimaging and Detection Applications

**DOI:** 10.3390/molecules27238421

**Published:** 2022-12-01

**Authors:** Yufei Yang, Fucheng Gao, Yandong Wang, Hui Li, Jie Zhang, Zhiwei Sun, Yanyan Jiang

**Affiliations:** Key Laboratory for Liquid−Solid Structural Evolution and Processing of Materials, Ministry of Education, Shandong University, Jinan 250061, China

**Keywords:** fluorescent organic small molecules, bioimaging, detection, recognition mechanisms, fluorescent organic nanoparticles

## Abstract

The activity levels of key substances (metal ions, reactive oxygen species, reactive nitrogen, biological small molecules, etc.) in organisms are closely related to intracellular redox reactions, disease occurrence and treatment, as well as drug absorption and distribution. Fluorescence imaging technology provides a visual tool for medicine, showing great potential in the fields of molecular biology, cellular immunology and oncology. In recent years, organic fluorescent probes have attracted much attention in the bioanalytical field. Among various organic fluorescent probes, fluorescent organic small molecule probes (FOSMPs) have become a research hotspot due to their excellent physicochemical properties, such as good photostability, high spatial and temporal resolution, as well as excellent biocompatibility. FOSMPs have proved to be suitable for in vivo bioimaging and detection. On the basis of the introduction of several primary fluorescence mechanisms, the latest progress of FOSMPs in the applications of bioimaging and detection is comprehensively reviewed. Following this, the preparation and application of fluorescent organic nanoparticles (FONPs) that are designed with FOSMPs as fluorophores are overviewed. Additionally, the prospects of FOSMPs in bioimaging and detection are discussed.

## 1. Introduction

The key substances inside the human body, including metal ions [1], biological small molecules [2,3], reactive oxygen species [4,5,6] and reactive nitrogen [7,8], are closely related to biological events, regulating physiological functions and metabolism. For instance, metal ions (e.g., Fe^3+^, Na^+^, Al^3+^ and Mg^2+^) play important regulatory roles in metabolism and osmolality [9,10,11]. Biological small molecules, such as homocysteine (Hcy), cysteine (Cys) and glutathione (GSH) participate in metabolism and redox reactions [12,13,14]. The content of reactive oxygen species and reactive nitrogen reflects many pathological states, especially the development of cancer [15,16,17,18]. Therefore, monitoring the levels of these substances is helpful for understanding human health status.

With the development of in vivo imaging technology, more and more tracer techniques have been applied in biomedical research. Traditional tracing methods are usually required to remove tissues from the body, which cannot realize the real-time dynamic monitoring of the target substances. Currently, the commonly used in vivo tracer techniques include radionuclide imaging, magnetic resonance imaging (MRI), optical imaging and so on [19]. Radionuclide imaging can achieve quantitative and localization analysis of cells, but it has the shortcomings of low spatial resolution and the need of expensive equipment [20,21]. MRI with high spatial and temporal resolution enables monitoring of changes in cellular function, but this technique faces the problem of long imaging time [22]. In vivo luminescence imaging is a non-invasive technology, including bioluminescence imaging (BLI) and fluorescence imaging, mainly used to study gene expression and cell activity. Compared with BLI, the signal of fluorescence imaging is stronger and the detection accuracy is higher [23,24,25]. At present, in vivo fluorescence imaging has become a hotspot in biomedical research due to its advantages of low toxicity, high spatiotemporal resolution and utilization of an inexpensive instrument [26,27,28]. For example, fluorescent dye indocyanine green (ICG) imaging has been used in a variety of abdominal surgery applications, such as lymph node localization, ureteral detection and tumor identification [29]. In recent years, some fluorescent probes with high specificity have also been developed and used for in vivo imaging [30,31,32].

To date, many types of fluorescent probes, such as fluorescent organics [33,34,35,36], fluorescent proteins [37], inorganic nanoparticles [38,39] and semiconductor polymer nanoparticles [40,41] have been developed and widely used in bioimaging. Fluorescent proteins can be generated by cells themselves through genetic engineering, which is convenient for in vivo imaging. However, they are hard to metabolize in the body. Inorganic nanoparticles have good spectral properties and light stability, but the biological toxicity limits their application in bioimaging. Semiconductor polymer nanoparticles have high fluorescence brightness, but slow metabolism causes them to accumulate in the liver. Comparatively, fluorescent organic small molecule probes (FOSMPs) attracted more attention because of their controllable synthesis, stable luminescence, good biocompatibility, sensitive response and high signal-to-noise ratio [42,43]. When applied to imaging, the photochemical properties of FOSMPs are generally more stable than other types of probes. Additionally, small molecules also help to achieve higher fluorophore density and spatial resolution. Therefore, a large number of FOSMPs have been designed and applied to detect substances in various matrix [44,45,46,47,48,49,50,51]. Fluorescent organic nanoparticles (FONPs), which were designed with FOSMPs as fluorophores, show the ability to build a multifunctional biosensing platform through surface functionalization and drug encapsulation [52]. A summary of the progress on the applications of FOSMPs in bioimaging and detection is of significance for the development of new diagnostic tools. As far as we know, there are no special reviews focusing on this field. This review focuses on the application of FOSMPs and FONPs in bioimaging and detection, including the fluorescence mechanism, detection principle, representative examples and development prospect. This review aims to provide a reference for the development of new tools and strategies for in vivo biosensing of key substances by using FOSMPs.

## 2. FOSMPs for Bioimaging and Detection

**Figure 1:**
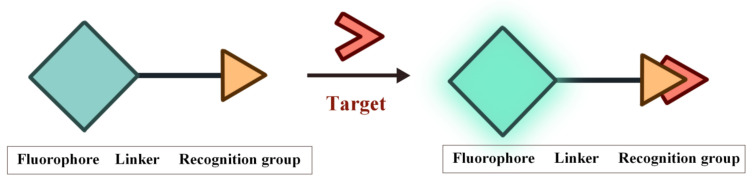
The structure of FOSMPs and its recognition of the target.

Fluorescence is the phenomenon that electrons return from the first singlet state to the ground state with concomitant energy release in the form of light. The characteristics of the fluorescent probes such as excitation and emission wavelength, intensity, lifetime and polarization are easily influenced by the environment conditions, thereby providing sensitive signals for the tracking and monitoring of analytes [53,54,55,56,57,58]. The structure of FOSMPs is mainly composed of three parts: the recognition group, the fluorophore, and the linking group (linker) (Figure 1). The recognition group endows the fluorescent probe with detection selectivity. The fluorophore provides the signal response when the probe recognizes the target. The linker connects the recognition group and the fluorophore and is not necessary for the fluorescent probe.

**Figure 2:**
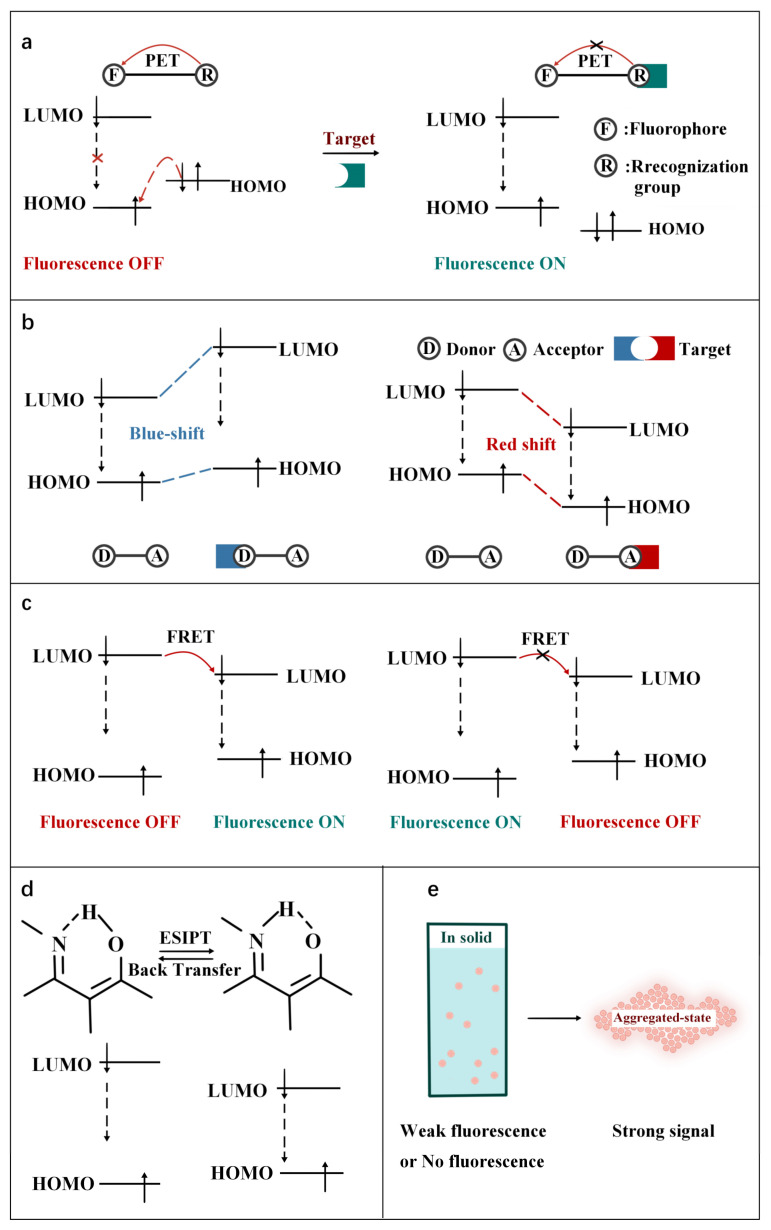
Schematic illustration of the principles of (**a**) PET, (**b**) ICT, (**c**) FRET, (**d**) ESIPT and (**e**) AIE.

As shown in Figure 2, according to the interaction manner between FOSMPs and targets, the recognition mechanisms can be divided into five types: photo-induced electron transfer (PET), intramolecular charge transfer (ICT), fluorescence resonance energy transfer (FRET), excited state intramolecular proton transfer (ESIPT) and aggregation-induced emission (AIE). Based on these recognition mechanisms, various FOSMPs for bioimaging and detection have been developed, as listed in Table 1. In the following, the applications of FOSMPs in bioimaging and analysis based on these recognition mechanisms are reviewed.

### 2.1. PET FOSMPs

**Figure 3:**
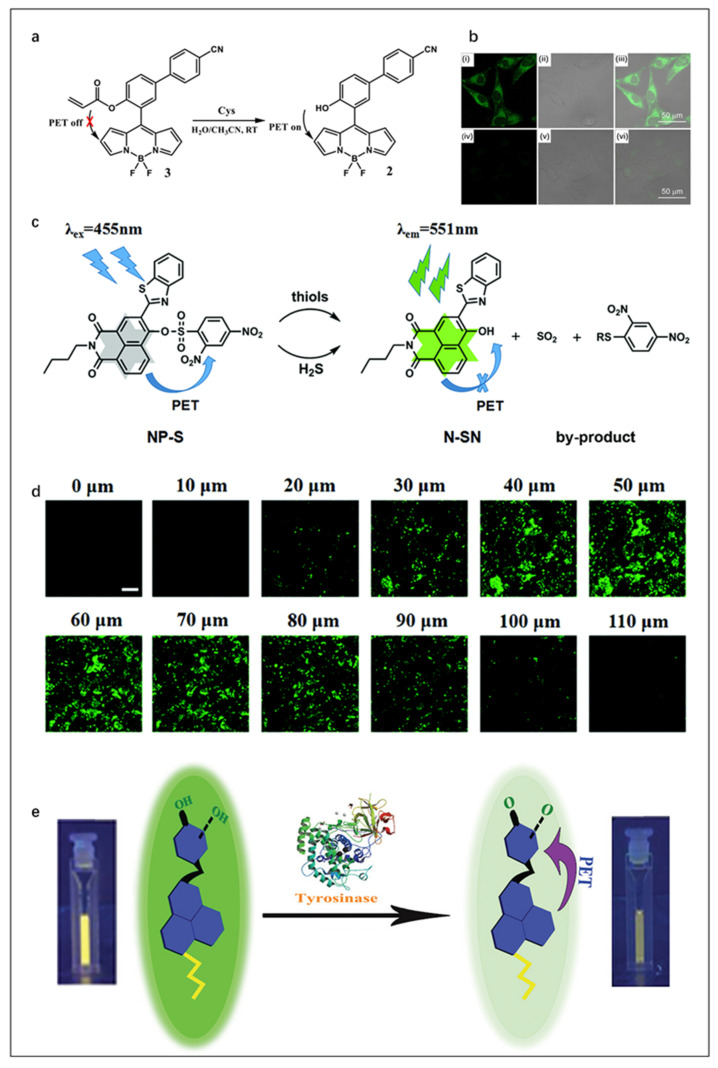
(**a**) The concept of a probe for Cys detection. (**b**) Confocal microscopy images of Cys detection in live A375 cells using probe. (**i**) Cells incubated with probe (20 μM) for 30 min at 37 °C, (**ii**) bright field of (**i**), (**iii**) merge of (**i**,**ii**), (**iv**) probe-treated A375 cells further incubated with Cys (1 mM) for 30 min, (**v**) bright field of (**iv**), (**vi**) merge of (**iv**,**v**). Reproduced with permission from Ref. [59], copyright 2016, Elsevier B.V. (**c**) The chemical structures and the sensing mechanism of the NP-S for detecting thiols and H_2_S. (**d**) TP imaging for mouse liver slices (Scale bar: 200 mM). Reproduced with permission from Ref. [60], copyright 2022, Royal Society of Chemistry. (**e**) Schematic illustration of the detection principle of tyrosinase. Reproduced with permission from Ref. [61], copyright 2020, Royal Society of Chemistry.

The mechanism of PET is displayed in Figure 2a. The excited electrons on the LUMO orbital of the fluorophore cannot normally transition to its HOMO orbital when the HOMO orbital or LUMO orbital of the recognition group is between the HOMO and LUMO orbitals of fluorophore. As a result, the fluorescence of fluorophore is quenched. When binding to the target, the HOMO and LUMO orbitals of recognition group changes so that the excited electrons on the LUMO orbit of the fluorophore can normally transition to its HOMO orbital, which cuts off the PET and recovers the fluorescence emission of fluorophore [83]. Herein, several typical PET FOSMPs are introduced [59,60,61,62]. For instance, Fan et al. [59] reported a PET-based strategy for Cys detection (Figure 3a). The addition of Cys turned on the PET, and the fluorescence intensity of the probe was linearly correlated with the concentration of Cys. When 250 μM of Cys was added, the fluorescence intensity was almost completely quenched, with a decrease of approximately 20-fold as compared with that of the free probe. Due to its low toxicity and high membrane permeability, this probe was successfully used for bioimaging (Figure 3b).

Xu et al. [60] synthesized a fluorescent probe named NP-S, with 2,4-dinitrobenzenesulfonyl (DNBS) as the recognition group and naphthalimide platform as the fluorophore (Figure 3c). By combining high resolution with strong tissue penetrating two-photon (TP) imaging technology, NP-S realizes high-precision detection of thiol and H_2_S in vivo. The TP imaging results of mouse liver slices showed that NP-S had good tissue imaging performance (Figure 3d).

In order to realize rapid detection at a nanomolar level, Kumar et al. [61] designed a fast fluorescence probe with good photostability and pH resistance for tyrosinase detection, which can detect tyrosinase as low as 2 nM immediately after the addition of tyrosinase (Figure 3e). This probe can achieve a two-fold decrease in fluorescence intensity within 5 min. The confocal images of B16F10 cells treated with probe tyro1 demonstrated the effectiveness of the probe in detecting tyrosinase in living cells. Moreover, its fast fluorescence kinetics also has guiding significance in screening the application of tyrosinase inhibitors.

PET-based fluorescent probes have high sensitivity. However, the false positive signals generated by the combination of protons with electron-rich recognition groups in the biological environment is still a challenge in designing a PET-based fluorescent probe suitable for a wide pH range.

### 2.2. ICT FOSMPs

**Figure 4:**
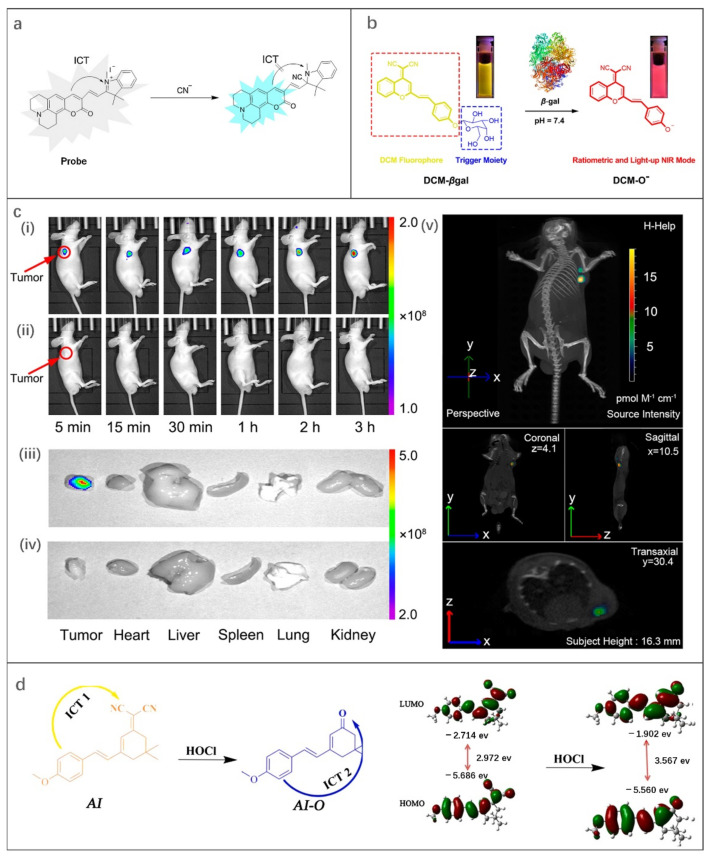
(**a**) The ICT mechanism of the probe. Reproduced with permission from Ref. [63], copyright 2015, Elsevier B.V. (**b**) The ICT mechanism of DCM-βgal. (**c**) (**i**,**ii**) In vivo imaging of β-gal activity in tumor-bearing nude mice after tumor injection, (**iii**,**iv**) fluorescence images of the main internal organs after anatomy, (**v**) three-dimensional in vivo imaging of β-gal activity in tumor-bearing nude mice after tumor injection of DCM-βgal for 3 h. (**i**,**iii**,**v**) Avidin-β-gal (100 μg) in PBS was intravenously injected into LoVo-implanted mice, and after 18 h DCM-βgal was then injected into the mice. (**ii**,**iv**) Tumor-bearing mice were not pretreated with avidin-β-gal before injection of DCM-βgal acting as the control. Reproduced with permission from Ref. [64], copyright 2022, American Chemical Society. (**d**) Theoretical calculation of HOMO/LUMO energy gaps of AI and AIO. Reproduced with permission from Ref. [67], copyright 2020, Elsevier B.V.

As depicted in Figure 2b, the mechanism of ICT is the change of HOMO and LUMO energy gaps of excited fluorophores caused by intramolecular charge transfer. Typically, a pair of electron donor and acceptor are connected by spacer inside the molecule. When the fluorophore molecule is exposed to the target, the degree of charge transfer changes, resulting in a “turn-on” effect response of fluorescence or a shift in wavelength [84]. Up to now, some FOSMPs based on ICT mechanism have been designed [63,64,65,66,67,68]. For example, Xiong et al. [63] designed an ICT-based probe for CN^-^ detection. The CN^-^ nucleophilic addition at the polarized C=N bond of the probe blocked the ICT process and turned on the fluorescence response, resulting in enhanced fluorescence of the green channel and blue shift of the UV-visible spectrum (603 nm to 440 nm) (Figure 4a). The probe proved to be capable of visualizing imaging in HepG2 cells.

β-gal is an important biomarker of aging and primary ovarian cancer [85]. Gu et al. [64] reported an in vivo NIR probe targeting β-gal and DCM-βgal, which achieved real-time in vivo 3D imaging of β-gal for the first time. In the presence of β-gal, the sugar portion of DCM-βgal was cleaved, thereby turning on the NIR fluorescence response (Figure 4b). This probe enabled accurate detection of β-gal with a fluorescence plateau lasting 35 min. The intensity of the emission spectrum at 685 nm increased linearly with the increase of the concentration of β-gal in the range of 0−12 U mL^−1^, and the LOD was 1.7 × 10^−4^ U mL^−1^. Human embryonic kidney cells (293T cells) were selected as a model cell line to achieve β-gal overexpression by gene transfection and to obtain quantitative ratio imaging of endogenous β-Gal activity. In addition, an IVIS spectral CT imaging system was used to obtain real-time, high-resolution 3D fluorescence images in tumor-bearing mice (Figure 4c). The fluorescence could be observed within 5 min of injection.

Shi et al. [67] synthesized a new type of HOCl fluorescent probe named AI with anisaldehyde as the donor and dicyanoisophorone as the acceptor. In the presence of HOCl, the intramolecular electrotraction group changed from the CN group to the ketone group with a lower electronegativity (Figure 4d), causing the blue shift of absorption and emission wavelength. In addition, AI can also be used to detect endogenous and exogenous HOCl acid in HeLa cells. Based on the results of cell experiments, the practicability of artificial intelligence in vivo imaging was further performed, and a strong fluorescence signal was observed by subcutaneous injection in mice.

Fluorescent probes based on the ICT mechanism is widely used in the fields of bioimaging and detecting due to its simple principle [86,87,88]. However, if the electron donor binds to the electron-withdrawing targets (EWTs), the electron transfer of the ICT can be suppressed and the fluorescence is thus quenched [89].

### 2.3. FRET FOSMPs

A FRET system consists of donors and acceptors. The phenomenon of energy transfer from the excited donors to the ground state acceptors in a limited distance (10–100 Å) is called FRET. (Figure 2c). FRET can be turned on or off by changing the structure of the acceptors or the distance between the donors and the acceptors [90].

**Figure 5:**
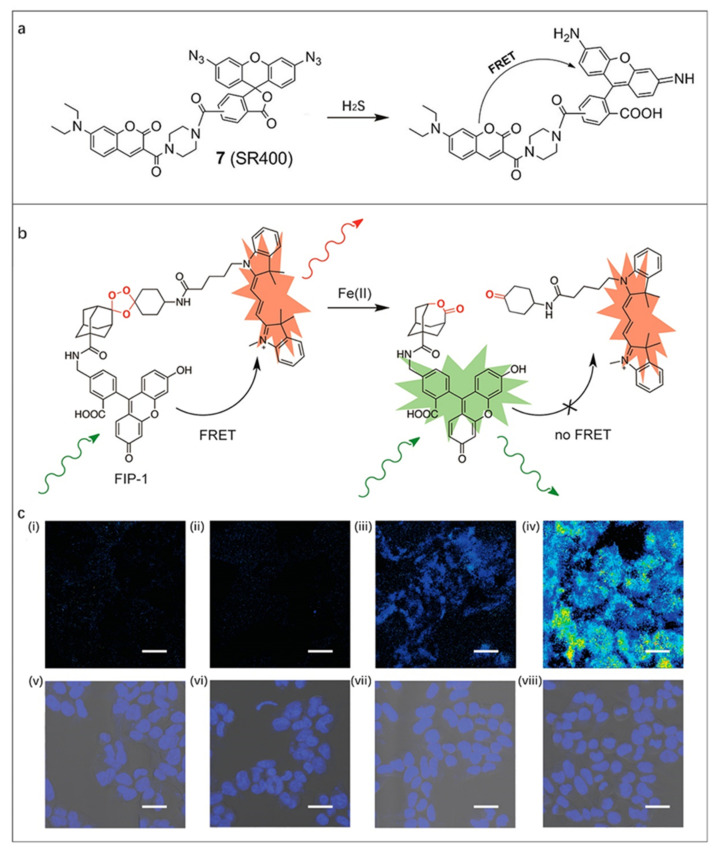
(**a**) The recognition mechanism of SR400 towards H_2_S. Reproduced with permission from Ref. [69], copyright 2022, Springer Nature. (**b**) Design of FRET Fe(II) probe FIP-1. (**c**) Representative confocal microscopy images of live HEK 293T cells loaded with FIP-1. Cells treated with (**i**) 1 mM bathophenanthroline disulfonate for 9.5 h, (**ii**) 250 μM deferoxamine for 9.5 h, (**iii**) vehicle for 90 min, (**iv**) 100 μM ferrous ammonium sulfate for 90 min. (**v**–**viii**) Brightfield images of (**i**–**iv**) overlaid with Hoechst stain. Reproduced with permission from Ref. [71], copyright 2022, American Chemical Society.

In recent years, significant progress has been made in the design of FOSMPs based on FRET mechanism [69,70,71,72,73]. Many FRET-based FOSMPs have been prepared and successfully used in the analytical chemistry field. Typically, Wei et al. [69] designed two ratiometric fluorescent probes (SR400 and SR550) using azido groups as reaction groups for detection of H_2_S in live cells, as shown in Figure 5a. The exposure of fluorescent probe to H_2_S caused the reduction of azido groups, triggering the acceptor from the closed non-fluorescent lactone structure converted to the open fluorescent structure and turning on the fluorescence response. The fluorescence intensity of the donor was weakened while that of the acceptor can be observed. Other reactive sulfur species that cannot trigger the response of the probe demonstrated the high detection selectivity of the probe for H_2_S. The designed ratiometric fluorescent probe realized the imaging of H_2_S in human embryonic kidney 293 cells, which is meaningful for guiding the application of H_2_S-releasing drugs.

Iron is the most abundant transition metal in the human body, and its level imbalance is associated with many diseases including cardiovascular diseases and cancer [91]. The intrinsic fluorescence quenching effect of Fe(II) poses a challenge to the design of fluorescent probes towards Fe(II). Based on this, Aron et al. [71] designed a reactivity-based ratiometric fluorescent probe FIP-1 to perform the imaging and detection of Fe(II) in living systems. The problems of ion interference and Fe(II)-dependent fluorescence quenching in biological environments were solved. Specifically, Fe(II) induced fluorescence response by blocking the FRET via cracking the peroxy bridging group between the donor and the acceptor inside the probe molecule (Figure 5b). Fluorescence images of live HEK 293T cells loaded with FIP-1 showed that the cells treated with Fe(II) exhibited reduced FRET effects compared with control cells, demonstrating the potential of FIP-1 in biological applications (Figure 5c).

Despite the above progress, emission spectral overlap between donor and acceptor reduces the accuracy of the fluorescence signal measurements. The fluorescence lifetime of the donor is shortened in the case of FRET [92]. Therefore, combining fluorescence lifetime imaging microscope (FLIM) with FRET can avoid the correction by using complex mathematical algorithms and provide higher temporal and spatial resolution [93]. Moreover, the coupling of FRET-FLIM with other advanced optical and mathematical technologies (such as adaptive optics [94] and unsupervised analysis [95]) may facilitate more comprehensive and precise monitoring of analytes.

### 2.4. ESIPT FOSMPs

ESIPT mechanism was first proposed by Weller in the 1950s [96], and it is based on proton transfer within a molecule from the relatively acidic region (e.g., hydrogen bond donor, −OH, −NH_2_) to the basic region (e.g., hydrogen bond acceptor, =N−, C=O). In most cases, there is isomerization between ketones and enols in the ESIPT system, which changes the energy gap between the HOMO and LOMO orbitals of fluorophore and causes a shift in the fluorescence emission wavelength (Figure 2d). The shift in the fluorescence emission wavelength results in double fluorescence, characterized by shorter wavelengths for enol-like structures and longer wavelengths for ketone-like structures. Till now, many ESIPT-based fluorescence sensors have been designed for biocomponent analysis [74,75,76].

**Figure 6:**
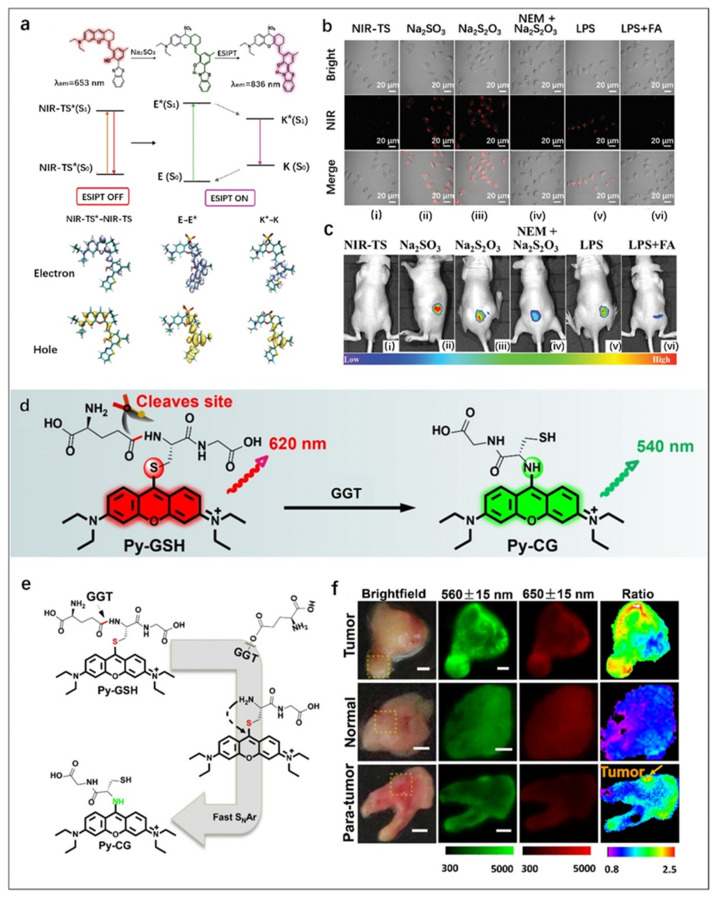
(**a**) Design of NIR-TS probe for SO_2_ detection. (**b**) NIR fluorescence imaging of SO_2_ (Na_2_SO_3_) in HeLa cells. (**i**) HeLa cells were incubated with the NIR-TS probe (10 µM) for 15 min. (**ii**) HeLa cells were pre-treated with the NIR-TS probe and then incubated with exogenous Na_2_SO_3_ (50 µM) for 10 min. (**iii**) HeLa cells were pretreated with the NIR-TS probe (10 µM), then incubated with Na_2_S_2_O_3_ (500 µM) for 30 min. (**iv**) NIR-TS probe-stained HeLa cells were incubated with 500 µM N-ethylmaleimide (NEM; thiol inhibitor) for 30 min, followed by Na_2_S_2_O_3_ for another 30 min. (**v**) HeLa cells were treated with the NIR-TS probe, then incubated with 1 µg mL^−1^ lipopolysaccharide. (**vi**) HeLa cells were incubated with the NIR-TS probe, then FA (200 µM) and lipopolysaccharide (1 µg mL^−1^) were added. (**c**) NIR fluorescence imaging of Na_2_SO_3_ in mice. (**i**) NIR-TS probe-treated (10 µM) mice. (**ii**,**iii**,**v**) NIR-TS probe-treated (10 µM) mice were also injected with Na_2_SO_3_, Na_2_S_2_O_3_ and LPS, respectively. (**iv**) As a control of (**iii**), mice were treated with NEM, followed by Na_2_S_2_O_3_ and the NIR-TS probe. (**vi**) As a control of (**v**), mice were treated with LPS, followed by FA and NIR-TS probe. Reproduced with permission from Ref. [76], copyright 2021, Royal Society of Chemistry. (**d**) Design of ratiometric fluorescence probe Py-GSH for GGT detection. (**e**) Responsive mechanism of Py-GSH to GGT. (**f**) Fluorescence images of the human tissues after stain with 10 μM Py-GSH saline for 10 min. In fluorescence tissue imaging, the emission channels at 560 ± 15 nm (Green channel) and 650 ± 15 nm (Red channel) were collected. In ratiometric imaging, the ratio of emission intensity at 560 ± 15 nm to 650 ± 15 nm was chosen as the detected signal. Λex = 488 nm. Scale bar: 2 mm. Reproduced with permission from Ref. [75], copyright 2022, Ivyspring International Publisher.

Endogenous SO_2_ mediates many physiological processes, such as blood pressure and cardiac contractility, and plays an important role in maintaining redox homeostasis in organisms [97,98]. According to ESIPT mechanism, Ren et al. [76] designed a FOSMP with 2-(2′-hydroxyphenyl) benzothiazole as the fluorophore, as shown in Figure 6a. In the presence of SO_2_, the electron distribution of NIR-TS changed, resulting in a red shift of the fluorescence emission wavelength with NIR-enhanced emission at 836 nm and a large Stokes shift of 286 nm. The fluorescence signal stabilized in 10 s, and the fluorescence intensity was up to 30 folds that of the probe itself. NIR-TS has been successfully used for fluorescence imaging in Hela cells (Figure 6b) and mice (Figure 6c) with good mitochondrial targeting. This work provides a new idea for designing long wavelength ESIPT fluorescent probes.

Zhou et al. [75] developed a highly sensitive ratiometric fluorescent probe (Py-GSH) for the sensing of GGT, a biomarker of ovarian cancer (Figure 6d). The presence of GGT resulted in γ-glutamyl cleavage and subsequent intramolecular rearrangement, finally generating a new fluorescent molecule Py-GG (Figure 6e). It could be observed from the fluorescence images of the probe in human specimens that the green/red fluorescence ratio in tumor tissue was significantly higher than that in normal tissue (Figure 6f). These different ratios could provide a strong basis for the diagnosis of tumor lesions, which proved that this biodetection platform with high sensitivity and low toxicity had promising application prospects in rapid cancer diagnosis and surgical navigation during tumor resection.

Among all intramolecular photophysical reactions, ESIPT has the most significant Stokes shift [99]. However, strong hydrogen-bonded solute-solvent environments can reduce ESIPT efficiency and significantly retard ESIPT kinetics due to reliance on intramolecular proton transfer [100].

### 2.5. AIE FOSMPs

The concept of AIE was first proposed by Benzhong Tang in 2001 [101]. Materials with AIE effect have a unique luminescence characteristic. As depicted in Figure 2e, the molecular system does not emit fluorescence or emit weak fluorescence in the solution condition due to the free rotation of the chemical bonds. However, enhanced fluorescence is generated in the aggregation state because the rotation is inhibited. This phenomenon overcomes the aggregation-caused quenching effect of traditional fluorescent materials [102]. Based on this mechanism, many fluorescent probes have been designed [77,78,79].

**Figure 7:**
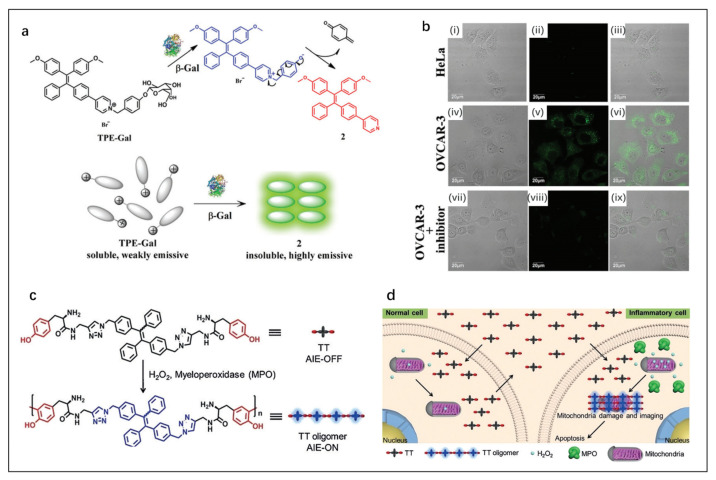
(**a**) Schematic representation of light-up sensing of β-gal. (**b**) Confocal fluorescence microscopy images of HeLa and OVCAR-3 cells incubated with TPE-Gal (10 mM) for 40 min: (**i**–**iii**) HeLa cells, (**iv**–**vi**) OVCAR-3 cells, and (**vii**–**ix**) OVCAR-3 cells pretreated with an inhibitor (50 mM) (λex = 405 nm). Reproduced with permission from Ref. [77], copyright 2022, Royal Society of Chemistry. (**c**) Peroxidase-catalyzed oligomerization of TT in the presence of H_2_O_2_. (**d**) Selective imaging and inhibition of inflammatory cells after incubation of co-cultured cells with TT. Reproduced with permission from Ref. [78], copyright 1999–2022, John Wiley & Sons, Inc.

Jiang et al. [77] reported a tetrabenyl (TPE)-based fluorescent probe TPE-Gal for β-gal detection. As shown in Figure 7a, by cleaving intramolecular β-galactoside, β-gal guided the probe molecule to form a phenolic intermediate, and the intermediate will spontaneously undergo 1,6-elimination of p-quinone-methide to obtain a poorly water-soluble product 2. AIE effect occurred in the aggregated state of product 2, and the fluorescence intensity gradually increased and reached a plateau in 9 min. TPE-Gal showed very weak fluorescence emission in HeLa cells but showed strong fluorescence in OVCAR-3 cells from human ovarian cancer patients, indicating the overexpression of endogenous β-gal in tumor cells (Figure 7b). These results demonstrated the potential of TPE-Gal in cancer diagnosis.

Cheng et al. [78] designed a new therapeutic multifunctional AIE probe named TT. When exposed to H_2_O_2_ and overexpressed myeloperoxidase in inflammatory cells, TT can crosslink into hydrophobic aggregates, activating the AIE (Figure 7c). In addition, a large number of TT aggregates induced mitochondria damage and inflammatory cell apoptosis but were harmless to normal cells (Figure 7d). Cell imaging proved that TT can distinguish inflammatory cells from normal cells and has a selective cell inhibition effect. TT is a powerful tool for accurate inflammation detection and treatment.

Because the triggering of AIE effect requires a high concentration of local molecules, reducing cytotoxicity is a design consideration. At the same time, the ubiquitous conjugated phenolic rings in AIE probes may lead to intracellular distribution and metabolic problems [103].

### 2.6. Multi-Mechanism FOSMPs

Compared to single sensing mechanism-based FOSMPs, multiple sensing mechanism-based FOSMPs can achieve signal amplification and diversification, which is conducive to realize the tracking of a variety of analytes and to improve the sensitivity of the detection [80,81,82].

**Figure 8:**
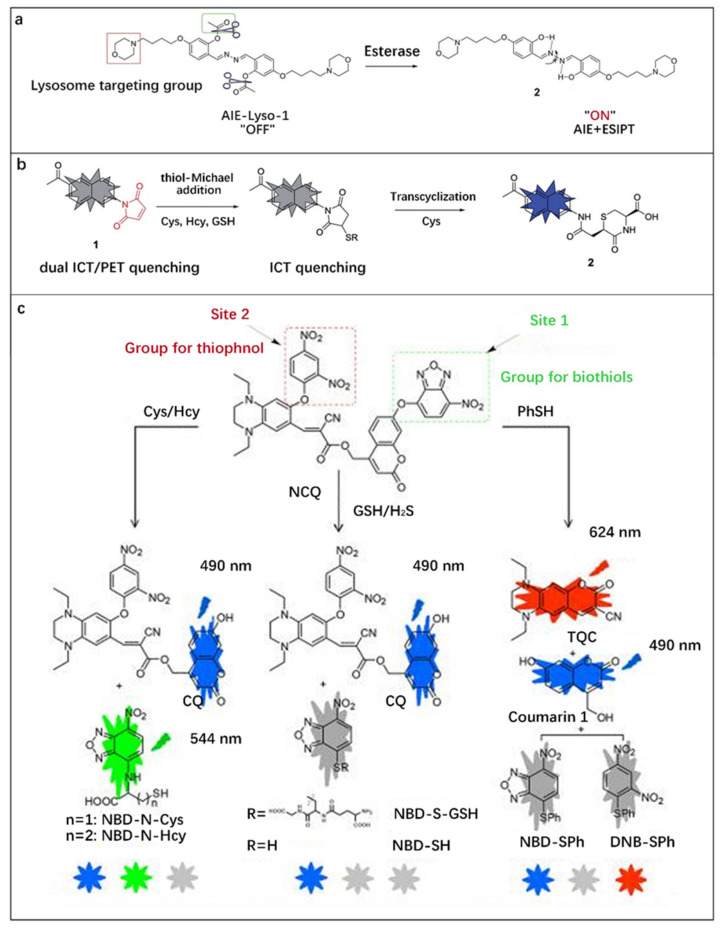
(**a**) Design of AIE-Lyso-1 for specific detection of lysosomal esterase. Reproduced with permission from Ref. [80], copyright 2014, Royal Society of Chemistry. (**b**) Design principle of probe 1 for selective detection of Cys. Reproduced with permission from Ref. [81], copyright 2018, Royal Society of Chemistry. (**c**) Sensing Mechanism of NCQ for distinguishing Cys/Hcy, GSH/H_2_S and thiophenol. Reproduced with permission from Ref. [82], copyright 2022, American Chemical Society.

Gao et al. [80] reported a fluorescent probe named AIE-Lyso-1 with “AIE + ESIPT” characteristics for in situ visualization of lysosomal esterase activity and motility (Figure 8a). The probe molecule consists of lysosome-targeted morpholine, esterase-reactive acetoxyl, and salicyladazine fluorophore. The free rotation of the probe molecule resulted in quenching of the salicyladazine fluorophore. After reacting with esterase, the acetyl group was cleaved and the product molecule 2 was generated. The formation of intramolecular hydrogen bond activated the ESIPT and AIE reactions of molecule 2 at the same time. Real-time imaging of MCF-7 cells proved that AIE-Lyso-1 could be used for in situ monitoring of lysosomal esterase activity. In addition, this probe has the advantages of no self-quenching and good luminescence in lysosomal profiling. Furthermore, this dual-mechanism sensing is conducive to the formation of a large Stokes-shifted ESIPT emission that amplifies the response signal.

Biothiols with similar molecular structures, such as Cys, Hcy, GSH, H_2_S and PhSH play important roles in enzymatic catalysis, redox reactions, protein synthesis and other metabolic activities in the human body. The imbalance in the concentration levels of these substances is related to some diseases [104,105]. Therefore, it is essential to develop methods to distinguish them. Based on “ICT + PET” mechanisms, Chen et al. [81] reported a fluorescent probe for the high-sensitivity and selective differentiation of Cys from other biothiols. The maleimide group typically exhibited no or weak fluorescence due to the presence of an effective PET quenching pathway in the LUMO orbital. Upon thiol-michael addition, the above orbital was removed and PET quenching was eliminated. Meanwhile, EWTs-induced fluorescence quenching of the succinimide group at the 4-position still kept the molecule in a low fluorescence. Only Cys adducts can remove the ICT quenching by intramolecular transcycination reaction, and more than 3000-fold fluorescence could be achieved due to the elimination of the double quenching effect (Figure 8b).

In order to achieve simultaneous tracking of Cys/Hcy, GSH/H_2_S, and PhSH in living cells, Yang et al. [82] integrated two sensing groups (2, 4-dinitrobenzene (DNB) and 7-nitro-1,2, 3-benzooxadiazole (NBD)) and two fluorophores into a fluorescent probe molecule (NCQ) through ether bonds, as shown in Figure 8c. After the reaction between active site 1 and Cys/Hcy, GSH/H_2_S and PhSH, respectively, NBD-S-Cys/Hcy with green fluorescence and NBD-S-GSH/NBD-SH and NBD-SPh without fluorescence were obtained. Active site 2 is active towards PhSH and inert towards the other four molecules. PhSH could break the ether bonds of DNB and NBD, releasing blue-emitting coumarin 1 and then forming red-emitting TQC. The detection can be realized by monitoring the combination of three-color fluorescence signals (blue-green-red). The detection limits of NCQ for Cys, Hcy, GSH, H_2_S and PhSH were 0.57, 0.65, 0.49, 0.52 and 0.34 μM, respectively.

## 3. FOSMPs-Based FONPs for Bioimaging and Detection

Compared with FOSMPs, FONPs have larger surface area, better biodegradability and greater resistance to photobleaching [106,107]. FONPs-based multifunctional biodetection platforms can be constructed through drug encapsulation and surface modification of targeting groups. This section briefly describes the preparation and applications of FOSMPs-based FONPs in bioimaging and detection.

**Figure 9:**
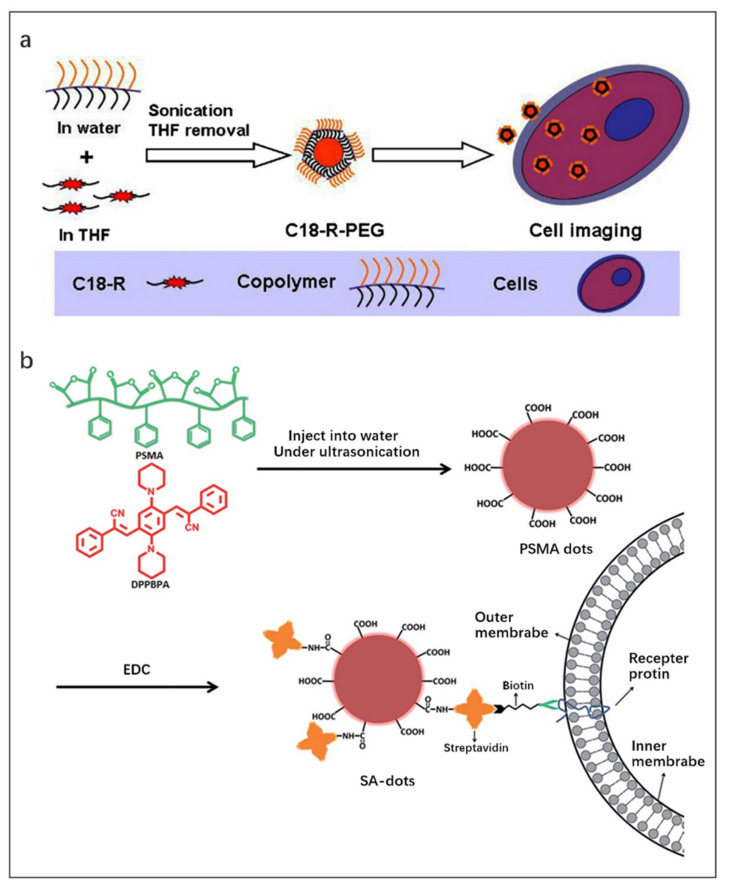
(**a**) Schematic illustration of the formation of C18-R-PEG FONPs via self-assembly of C18-R and synthetic copolymers and their utilization in cell imaging. Reproduced with permission from Ref. [108], copyright 2013, Published by Elsevier B.V. (**b**) Schematic illustration of the preparation of SA-dots via EDC-catalyzed coupling and their subsequent cell marking. Reproduced with permission from Ref. [109], copyright 2015, Royal Society of Chemistry.

Zhang et al. [108] reported a strategy to prepare FONPs by encapsulating the fluorophore C18-R in synthetic copolymer matrices (Figure 9a). First, the copolymer aqueous solution was added to the C18-Rd THF solution under sonication. In the process of removing THF, the hydrophobic segment of the copolymer wrapped C18-R through hydrophobic interaction to form a “core-shell” structure, and finally the C18-R-PEG FONPs were obtained. CLSM images and cell uptake experiments proved that the C18-R-PEG FONPs has good biocompatibility and can be used for bioimaging.

Enhanced fluorescence emission can be obtained by loading high concentrations of fluorophores into FONPs since AIE can occur at high concentrations of fluorophores. Zhang et al. [109] prepared near-infrared emitting AIE dots with a particle size of ~20 nm and a fluorescence quantum efficiency of 20% by using the amphiphilic polymer poly (styrene co maleic anhydride) (PSMA) as the co-encapsulation matrix and a novel small-molecule fluorophore (2Z,2′Z)-3,3′-(2,5-di(piperidin-1-yl)-1,4-phenylene)bis(2-phenylacrylonitrile) (DPPBPA) as the core (Figure 9b). The final product SA dots were obtained by modifying streptavidin on its surface to achieve specific binding to target cells. SA dots have been successfully used for fluorescence imaging of MCF-7 cell line benefit from their uniform size, stable luminescence and excellent biocompatibility.

**Figure 10:**
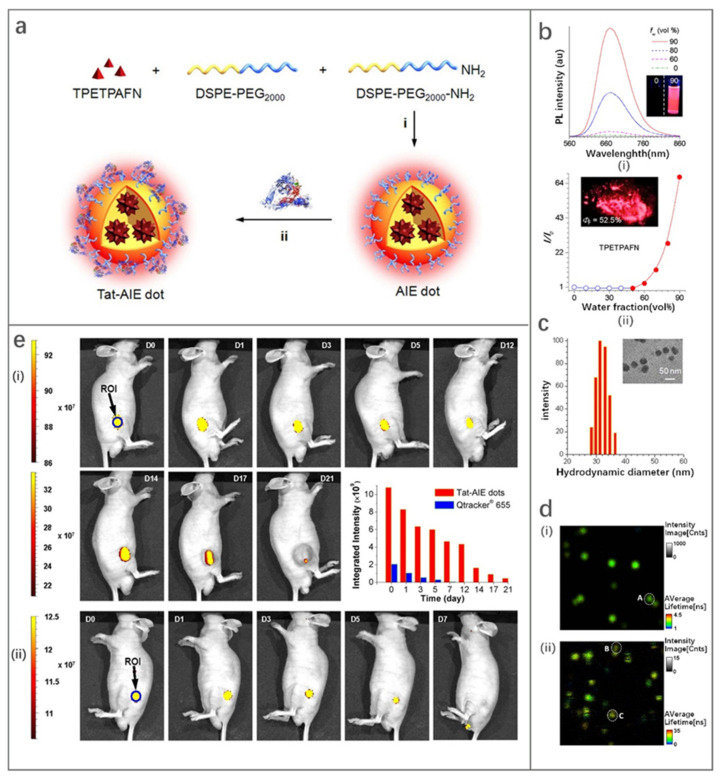
(**a**) Schematic illustration of fabrication of Tat-AIE dots. (**b**) (**i**) Photoluminescence (PL) spectra and (**ii**) I/I_0_ intensity of TPETPAFN in THF/water mixtures with different water fractions. (**c**) Particle size distribution and morphology of Tat-AIE dots. (**d**) Fluorescence lifetime imaging (FLIM, 5 × 5 µm^2^) results of (**i**) Tat-AIE dots and (**ii**) Qtracker^®^ 655. (**e**) In vivo fluorescence imaging of tumor cells by Tat-AIE dots. (**i**) Representative in vivo fluorescence images of the mouse subcutaneously injected with 1 × 10^6^ of C6 glioma cells after staining by 2 nM Tat-AIE dots. (**ii**) Data for Qtracker^®^ 655 obtained under similar conditions. The inset in the middle panel showed the integrated PL intensities of the regions of interest (blue circles) at the tumor sites from the corresponding images. Reproduced with permission from Ref. [111], copyright 2022, Springer Nature.

1,2-distearoyl-sn-glycero-3-phosphoethanolamine-N-(polyethylene glycol) (DSPE-PEG_2000_) is a typical package matrix that is widely used in the design of FONPs due to its good biocompatibility [110,111,112,113]. Transcription-AIE (Tat-AIE) dots prepared using DSPE-PEG_2000_ as the matrix was first reported as cell-tracing probes in 2013, showing brighter fluorescence, better fluorescence stability and cell-tracking ability than commercial quantum dot-based probes. In this work, Li et al. [111] encapsulated the fluorophore TPETPAFN by using a mixture of DSPE-PEG_2000_ and DSPE-PEG_2000_-NH_2_ (Figure 10a). Then, Tat-AIE dots were obtained by coupling AIE dots with cell penetrating peptide HIV-1 Tat. TPETPAFN has poor water solubility but is easily soluble in THF solution. The fluorescence of TPETPAFN was turned on when the THF/water volume ratio was 1:1 and the fluorescence intensity increased exponentially with the increase of the ratio of water. The fluorescence intensity of TPETPAFN showed a 70-fold enhancement at a 90% water volume fraction (Figure 10b). The hydrodynamic size and quantum yield of the as-prepared NIR-emitting Tat-AIE dots were ~30 nm and 24%, respectively (Figure 10c). Compared to commercial Qtracker^®^ 655, Tat-AIE dots displayed 10-fold stronger fluorescence intensity and better long-term tracing ability (Figure 10d). Tat-AIE dots could trace MCF-7 cells for 10–12 generations and trace C6 cells for 21 days in vivo (Figure 10e). This work shows the promise of FONPs as an ideal alternative to quantum dots in fluorescence imaging and non-invasive long-term cell tracing.

## 4. Conclusions and Perspectives

This review has summarized the applications of FOSMPs in bioimaging and detection relying on different mechanisms, including PET, ICT, AIE, FRET, ESIPT and their combinations. The performance of these sensing modalities was also discussed. FOSMPs based on different detection mechanisms show the advantages and deficiencies in fluorescent performance, sensitivity, accuracy, adaptability and biological toxicity. Compared to single-mechanism-based detection mode, multi-mechanism-based biosensing displays diversified fluorescence signals, leading to more precise detection. Furthermore, the synthesis and biological applications of FONPs designed with FOSMPs as fluorophores were also introduced. In short, FOSMPs have been widely used in the field of bioanalysis, greatly facilitating the diagnosis and treatment of various diseases.

Nevertheless, some improvements are still needed to facilitate the practical application of FOSMPs. First, the properties of FOSMPs are easily affected by the detection environment, which hinders their application in a complex physiological environment. The design of FOSMPs with a large Stokes shift, high fluorescence intensity, good fluorescence stability and low toxicity is an ongoing effort. In addition, many FOSMPs are designed based on irreversible chemical reactions. FOSMPs prepared by reversible chemical reaction should be further investigated to realize reversible and dynamic real-time detection of cell state. Furthermore, the design of FOSMPs with targeted delivery and signal amplification functions is also a promising direction in the future. Second, super-resolution microscope technology has been booming in recent years [114], and it provides more application scenarios for FOSMPs. FOSMPs with high quantum yield, long fluorescence life and multiple reaction sites are required to achieve resolution imaging at the molecular level and to monitor the dynamic changes of subcellular organelles by this novel technology. Third, the exploration of multi-mechanism detection modes should be strengthened to improve the sensitivity and reliability of bioimaging and detection. The combination of multiple mechanisms aims to construct sensors with large signal-to-noise ratios and strong fluorescent signals and can simultaneously trace multiple analytes. Fourth, matrices with good biocompatibility and stability and active surface groups for modifying FOSMPs should be explored to obtain multifunctional and practical FONPs. It is expected that the continuous development of FOSMPs and biosensing strategies based on FOSMPs will greatly facilitate the advancement of disease diagnosis techniques.

## Figures and Tables

**Table 1:** Fluorescence characteristic, recognition mechanisms, and applications of FOSMPs.

Probe	λex/λem (nm)	Mechanism	Analyte	Solvent System	Linear Detection Range	Limit of Detection (LOD)	Target	Ref.
Probe	500/525	PET	Cys	CH_3_CN-Water-HEPES buffer	0–100 μM	3.7 × 10^−2^ μM	A375 cells	[59]
NP-S	455/551	PET	H_2_S	PBS buffer-EtOH	30–300 μM	0.376 μM	Mouse liver slices	[60]
Tyro1, Tyro2	455/560	PET	Tyrosinase	Potassium phosphate buffer	-	-	B16F10 cells	[61]
NFP-G, NFP-A	440/541	PET	Formaldehyde (FA)	DMSO-PBS buffer	0–30 μM, 0–15 μM	1.2 μM, 0.18 μM	HepG-2 cells	[62]
Naphthalimide chromophore	400/502	ICT	CN^−^	HEPES buffer -CH_3_OH	0–15 μM	0.066 μM	HepG2 cells	[63]
DCM-β-gal	535/685	ICT	β-galactosidase (β-gal)	PBS buffer-DMSO	0–12 U/L	0.17 U/L	293T cells	[64]
DCDHF-Glu	510/613	ICT	γ-Glutamyltranspeptidase (GGT)	PBS buffer	0–40 U/L	0.0379 U/L	HepG2 cells, LO2 cells	[65]
SHC	370/540	ICT	hNQO1	PBS buffer	0–0.8 μM	0.0146 μM	HT-29 cells, MDA-MB-468 cells	[66]
AI	370/495	ICT	Hypochlorous acid (HOCl)	DMSO-PBS buffer	0–50 μM	0.84 μM	HeLa cells, Nude mice	[67]
P-ONOO^−^	365/480	ICT	ONOO^−^	DMSO-PBS buffer	0.429–3.0 µM	0.0104 µM	HeLa cells	[68]
SR400, SR550	400/525, 550/675	FRET	H_2_S	PBS buffer	-	-	HEK293 cells	[69]
CF	415/517	FRET	HNO	PBS buffer	0–100 μM	1.4 μM	HeLa cells	[70]
FIP-1	515/556	FRET	Fe(II)	HEPES buffer	-	-	HEK293 cells, MDAMB-231 cells	[71]
PNCy3Cy5	530/660	FRET	OONO^–^	Phosphate buffer-DMF	0–0.7 μM	6.5 × 10^−4^ μM	RAW264.7	[72]
FTR-βgal	450/540	FRET	β-gal	PBS buffer-EtOH	0–5 U/L	4.11 × 10^−8^ U/L	Hek293 cells	[73]
PPA	400/511	ESIPT	Palladium	CH_3_CN-PBS buffer	0–180 μM	0.028 μM	A549 cells	[74]
Py-GSH	488/545	ESIPT	GGT	PBS buffer	1–30 U/L	1 × 10^−2^ U/L	SKOV3 cells, HOSEpiC cells, Tumor-bearing mice, Human specimens	[75]
NIR-TS	550/836	ESIPT	SO_2_	Water	0.5–40 μM	0.067 μM	HeLa cells, Mice	[76]
TPE-Gal	344/512	AIE	β-gal	PBS buffer	8 × 10^−4^–4.8 × 10^−3^ U/L	3.3 × 10^−4^ U/L	HeLa cells, OVCAR-3 cells	[77]
TT	320/440–550	AIE	H_2_O_2_	DMSO-Water	-	-	RAW264.7 cells, HLF cells	[78]
QP-DNP	482/582	AIE	Hydrazine	DMSO-HEPES buffer	0–0.8 μM	0.055 μM	HeLa cells, Kunming mouse	[79]
AIE-Lyso-1	356/532	AIE/ESIPT	Esterase	DMSO–Water	100–500 U/L	2.4 U/L	MCF-7 cells	[80]
Probe 1	314/446	PET/ICT	Cys	PBS buffer	0.2–2.0 μM	1.4 × 10^−3^ μM	HeLa cells	[81]
NCQ	423/490, 544 423/490423/490, 624	Triple-emission	Cys/Hcy, GSH/H_2_S, thiopheno(PhSH)	PBS buffer-acetonitrile	0–10 μM/10 μM, 0–6 μM/8 μM, 0–70 μM	0.57 μM/0.65 μM, 0.49 μM/0.52 μM, 0.34 μM	HeLa cells	[82]

## Data Availability

Not applicable.
